# 伴t(14;19)(q32;q13)小B淋巴细胞增殖性疾病20例临床分析

**DOI:** 10.3760/cma.j.issn.0253-2727.2022.08.010

**Published:** 2022-08

**Authors:** 慧 杨, 睿 郭, 雨 时, 纯 乔, 雨洁 吴, 磊 范, 卫 徐, 扣荣 缪, 建勇 李, 海荣 仇

**Affiliations:** 南京医科大学第一附属医院，江苏省人民医院血液科，南京 210029 Department of Hematology, Jiangsu Province Hospital, The First Affiliated Hospital of Nanjing Medical University, Nanjing 210029, China

**Keywords:** 白血病，淋巴细胞，慢性, t（14;19）（q32;q13）, IGH/BCL3, 临床特征, 预后, Leukemia, lymphocytic, chronic, t(14;19)(q32;q13), IGH/BCL3, Clinical features, Prognosis

## Abstract

**目的:**

分析20例伴t（14;19）（q32;q13）的小B淋巴细胞增殖性疾病患者的临床特征及预后，以提高对此类少见病例的认识。

**方法:**

回顾性收集并分析2013年4月至2020年12月南京医科大学第一附属医院收治的20例伴t（14;19）（q32;q13）小B淋巴细胞增殖性疾病患者的临床资料，其中10例为慢性淋巴细胞白血病（CLL），10例为其他小B细胞恶性肿瘤。

**结果:**

男10例，女10例，中位年龄53.5（35～88）岁。所有患者均出现淋巴细胞绝对计数增多，19例出现淋巴结肿大，10例脾肿大。中位随访36（4～163）个月，3例死亡，11例患者至开始治疗时间（TTT）≤12个月。10例（50％）患者伴+12，2例（2/17,12％）伴13q−。t（14;19）与免疫球蛋白重链可变区（IGHV）无突变相关（17/19，89％），且存在IGHV4-39的偏向使用（7/17，41％）。应用二代测序在14例（14/17，82％）患者中检出一种或多种基因突变，共涉及25种基因异常，其中发生频率最高的是NOTCH1（35％），其次是SF3B1（24％）和KMT2D（18％）。对10例CLL患者分析发现，5例（50％）分期为Rai Ⅲ/Binet C期。在20例患者中，2例发生Richter转化。

**结论:**

伴t（14;19）异常的小B细胞恶性肿瘤显示出独特的临床生物学特征，常伴多种不良预后因素，倾向于具有侵袭性临床病程。

## Abstract (en)

**Objective:**

The clinical characteristics and prognosis of 20 patients with small B-lymphocyte proliferative disease with t(14;19)(q32; q13) were analyzed to improve the understanding of such rare cases.

**Methods:**

The clinical data of 20 patients with t(14; 19)(q32; q13) small B lymphocyte proliferative disease treated in the First Affiliated Hospital of Nanjing Medical University from April 2013 to December 2020 were retrospectively collected and analyzed. Among them, 10 cases were chronic lymphocytic leukemia (CLL) and 10 cases were other small B-cell malignancies.

**Results:**

Among the 20 cases, 10 were male and 10 were female, and the median age at diagnosis was 53.5（35–88）years old. All patients had absolute lymphocytosis, 19 patients had lymphadenopathy, and 10 patients had splenomegaly. With a median follow-up of 36（4–163）months, three patients died, and 11 patients had a time to treatment（TTT）≤12 months. Ten patients（50％）were accompanied by +12, two patients（2/17, 12％）were accompanied by 13q−. Moreover, we found that t（14;19）was associated with unmutated immunoglobulin heavy-chain variable（IGHV）somatic mutation（17/19, 89％）and a biased use of IGHV4-39（7/17, 41％）was observed. Next-generation sequencing detected one or more gene mutations in 14（14/17, 82％）cases and a total of 25 gene mutations had been revealed, of which the most frequent were NOTCH1（35％）, followed by SF3B1（24％）and KMT2D（18％）. For 10 CLL patients, five（50％）were defined as Rai Ⅲ/Binet C. It is noteworthy that among the 20 cases, two cases actually involved Richter transformation.

**Conclusion:**

Small B-cell malignant tumors with abnormal t（14;19）show unique clinical biological characteristics, often accompanied by a variety of adverse prognostic factors, and tend to have an aggressive clinical course.

B细胞淋巴瘤中涉及免疫球蛋白重链（IGH）基因座的染色体易位常具有重要意义，如伯基特淋巴瘤中的t（8;14）（q24;q32）（IGH/MYC）、套细胞淋巴瘤中的t（11;14）（q13;q32）（IGH/CCND1）和滤泡淋巴瘤中的t（14;18）（q32;q21）（IGH/BCL2）。t（14;19）（q32;q13）（IGH/BCL3）于1983年首次被描述，但其临床和病理意义至今仍未明确[1]。t（14;19）（q32;q13）是一种罕见的细胞遗传学异常，其在B淋巴细胞增殖性疾病（B-LPD）中发生率低于0.2％，最常报告于慢性淋巴细胞白血病（CLL）中[2]–[5]。本文系统分析南京医科大学第一附属医院8年间积累的20例患者的临床资料，为此类患者临床诊治及基础研究提供一定的参考。

## 病例与方法

1. 病例：回顾性分析2013年4月至2020年12月就诊于南京医科大学第一附属医院血液科，经CpG-寡脱氧核苷酸（CpG-ODN）培养后染色体核型具有t（14;19）（q32;q13）异常的病例。共发现20例患者，均为小B淋巴细胞增殖性疾病，其中10例参照中国慢性淋巴细胞白血病工作组（cwCLL）2018版CLL诊疗指南归类为CLL[6]。

2. 方法：收集并分析20例患者的临床资料，包括性别、年龄、Rai和Binet分期、症状及体征、实验室检查、免疫表型、细胞遗传学及分子学特征。

3. 随访：通过查阅门诊及住院病历或电话进行随访，随访截至2021年11月25日，中位随访时间36（4～163）个月。至开始治疗时间（TTT）定义为自诊断到开始接受治疗的时间间隔，总生存（OS）期定义为自诊断至任何原因导致死亡或随访截止的时间间隔。

4. 统计学处理：计量资料采用中位数（范围）进行描述，计数资料采用例数（百分比）描述。

## 结果

1. 临床特征：男10例，女10例，诊断时中位年龄53.5（35～88）岁。患者的诊断及临床特征见表1。所有患者均出现淋巴细胞绝对计数增多，中位淋巴细胞计数32.8（6.7～213.3）×10^9^/L，19例出现淋巴结肿大，10例脾肿大。在18例行EB病毒DNA检测的患者中，11例（61％）阳性。10例（50％）诊断为CLL，其临床及免疫表型特征见表2。10例CLL患者中位淋巴细胞计数58.8（6.7～213.3）×10^9^/L，中位年龄53（35～70）岁。2例患者年龄<40岁，分别为35岁和37岁，在CLL中不常见。CLL分期中5例为Rai Ⅰ/Binet B期，5例为Rai Ⅲ/Binet C期。有2例患者发生了Richter转化，分别为例12和例19。β_2_-微球蛋白（β_2_-MG）平均水平为3.85 mg/L。中位随访36个月，2例失访，18例患者中3例死亡，2例病情进展，11例患者TTT≤12个月。

**表1:** 20例伴t（14;19）的小B淋巴细胞增殖性疾病患者的临床特征

例号	年龄（岁）	性别	诊断及免疫表型	淋巴结肿大	脾肿大	EB病毒	至开始治疗时间（月）	总生存（月）	转归
1	54	男	CLL（CD5^+^CD10^−^）	有	无	阴性	21	40	CR
2	52	女	CLL（CD5^+^CD10^−^）	有	有	阳性	8	28	CR
3	37	男	CLL（CD5^+^CD10^−^）	有	无	阳性	6	60	进展
4	35	女	CLL（CD5^+^CD10^−^）	有	无	阴性	未治	32	随访
5	53	男	CLL（CD5^+^CD10^−^）	有	有	阳性	0	46	CR
6	48	女	CLL（CD5^+^CD10^−^）	有	有	阳性	12	163	死亡
7	61	男	CLL（CD5^+^CD10^−^）	有	有	阴性	18	69	稳定
8	58	女	CLL（CD5^+^CD10^−^）	有	无	NA	NA	NA	NA
9	53	女	CLL（CD5^+^CD10^−^）	有	无	阳性	12	21	PR
10	70	男	CLL（CD5^+^CD10^−^）	无	无	阳性	未治	10	随访
11	47	女	MCL（CD5^+^CD10^−^）	有	无	阴性	未治	53	进展
12	65	男	RS（CD5^+^CD10^−^）	有	无	阴性	0	NA	NA
13	49	男	SMZL（CD5^−^CD10^−^）	有	有	阳性	0	12	PR
14	88	女	B-LPD（CD5^−^CD10^−^）	有	无	阳性	0	8	死亡
15	57	男	B-LPD（CD5^+^CD10^−^）	有	有	NA	未治	18	随访
16	52	男	B-NHL（CD5^−^CD10^−^）	有	有	阴性	未治	24	随访
17	56	女	B-NHL（CD5^+^CD10^−^）	有	有	阴性	12	62	死亡
18	70	男	WM（CD5^+^CD10^−^）	有	有	阳性	37	85	稳定
19	51	女	RS（CD5^−^CD10^−^）	有	有	阳性	3	4	CR
20	76	女	B-NHL（CD5^+^CD10^−^）	有	无	阳性	6	44	稳定

注：CLL：慢性淋巴细胞白血病；MCL：套细胞淋巴瘤；RS：Richter综合征；SMZL：脾边缘区淋巴瘤；B-LPD：B淋巴细胞增殖性疾病；B-NHL：B细胞非霍奇金淋巴瘤；WM：华氏巨球蛋白血症；NA：未做或数据缺失；CR：完全缓解；PR：部分缓解

**表2:** 10例慢性淋巴细胞白血病（CLL）患者的临床及免疫表型特征

例号	WBC（×10^9^/L）	ALC（×10^9^/L）	HGB（g/L）	PLT（×10^9^/L）	β_2_-MG（mg/L）	流式细胞术抗原表达					CLL积分	CLL分期
						CD5	CD23	FMC7	CD79b/CD22	SIg		
1	13.9	12.4	131	175	2.34	阳性	阳性	阴性	弱表达	阳性	4	Rai Ⅰ；Binet B
2	241.8	213.3	77	114	2.90	阳性	阳性	阴性	弱表达	弱表达	5	Rai Ⅲ；Binet C
3	109.8	105.1	132	168	4.30	阳性	阳性	阴性	弱表达	弱表达	5	Rai Ⅰ；Binet B
4	11.6	6.7	125	388	3.98	阳性	阴性	阴性	弱表达	弱表达	4	Rai Ⅰ；Binet B
5	188.5	166.6	75	46	3.88	阳性	阴性	阴性	弱表达	阳性	3	Rai Ⅳ；Binet C
6	72.8	70.4	87	148	9.09	阳性	阳性	阴性	弱表达	阳性	4	Rai Ⅲ；Binet C
7	55.8	47.1	131	186	3.52	阳性	阴性	阴性	弱表达	阳性	3	Rai Ⅲ；Binet C
8	39.1	33.0	134	138	3.20	阳性	阳性	阳性	弱表达	阳性	3	Rai Ⅰ；Binet B
9	149.4	122.7	86	155	2.80	阳性	阳性	阴性	弱表达	阳性	4	Rai Ⅲ；Binet C
10	28.1	22.2	135	146	2.54	阳性	阳性	阴性	弱表达	阳性	4	Rai Ⅰ；Binet B

注：ALC：淋巴细胞绝对计数；β_2_-MG：β_2_-微球蛋白

2. 免疫表型结果：所有t（14;19）病例免疫表型均为CD19^+^CD20^+^CD10^−^，其中16例（80％）为CD5阳性。所有CLL病例均表达B系抗原且CD5阳性、CD22或CD79b弱阳性（表2）。7例（70％）CLL患者可检测到CD5和CD23共表达，另外3例为CD5^+^CD23^−^。7例（70％）CLL显示κ或λ表面免疫球蛋白（SIg）中或强表达。10例CLL中仅1例患者FMC7阳性。CLL评分使用改良的Matutes评分系统，3例（30％）CLL的评分为3分，主要与CD23阴性和SIg强表达有关。CLL积分虽未达到常规4分诊断标准（该标准可区分96.6％的CLL和非CLL），但结合CD79b弱表达、IGH/CCND1排除性检查阴性、CLL辅助免疫标志物检测（如CD43^+^、CD200^+^）、流式细胞术免疫表型特点及形态学特征，此3例患者最终诊断为CLL。

3. 细胞遗传学结果：20例染色体核型检出t（14;19）的病例均通过IGH和BCL3两种分离重排荧光原位杂交（FISH）探针进行了验证。多色FISH（M-FISH）探针还用于1例复杂核型t（14;19）病例（例3）的检测。所有20例患者均采用FISH检测IGH、BCL3、CEP12、IGH/CCND1，除3例患者细胞不足无法检测外，17例患者FISH探针加测TP53、ATM、MYB、D13S319，细胞遗传学结果详见表3。+12的阳性率为50％（10/20），TP53缺失率为24％（4/17）。MYB、D13S319和ATM基因缺失发生率分别为18％（3/17）、12％（2/17）和0（0/17）。FISH阳性细胞比例在8例患者（例3、5、10、13、14、15、18、19）中存在不同探针之间差异悬殊情况（根据差异≥30％标准计算），提示至少有两种不同大小的克隆存在。中期和间期细胞遗传学阳性检出率不一致是另一个值得关注的问题，如例14的t（14;19）阳性细胞，常规细胞遗传学阳性率为100％，而FISH检测阳性率仅为8％；例15常规细胞遗传学的阳性率为40％，而FISH的阳性率则为5％。上述差异主要与中期常规细胞遗传学运用CpG-ODN刺激培养细胞，恶性克隆异常增殖有关。此外，在5例患者中t（14;19）（q32;q13）是唯一的染色体异常，而在9例（45％）患者中，+12是最常见的伴随异常。

**表3:** 20例伴t（14;19）患者的荧光原位杂交及分子突变检测结果

例号	FISH检测结果								IGHV突变	基因使用	CLL/淋巴瘤基因突变检测
	IgH	+12	p53	11q−	13q−	6q−	t(11;14)	BCL3			
1	+	−	NA	NA	NA	NA	−	+	否	IGHV4-39*01	EP300^a^
2	+	−	−	−	−	−	−	+	否	IGHV3-9*01	CREBBP/NOTCH1^b^
3	+	+	+	−	−	−	−	+	NA	NA	POT1/NOTCH1^b^
4	+	−	−	−	−	−	−	+	否	IGHV4-39*01	−^b^
5	+	−	−	−	+	−	−	+	否	IGHV4-39*01	SF3B1/ATM/CREBBP/KMT2C/NOTCH1^a^
6	+	−	−	−	−	−	−	+	否	IGHV3-53*01	BRAF/FBXW7/SAMHD1/ARID1A^b^
7	+	−	−	−	−	−	−	+	否	IGHV4-34*01	−^b^
8	+	+	−	−	−	−	−	+	否	IGHV4-34*01	NA
9	+	+	−	−	−	−	−	+	否	IGHV4-39*01	KLF2/MYC/SF3B1/TNFAIP3/ATM/FAT1^a^
10	+	+	−	−	−	−	−	+	否	IGHV4-39*01	POT1^a^
11	+	−	NA	NA	NA	NA	−	+	否	IGHV4-34*01	−^a^
12	+	+	−	−	−	−	−	+	否	IGHV4-39*01	NA
13	+	−	−	−	+	+	−	+	否	IGHV3-53*01	SPEN/KLF2/MGA^a^
14	+	+	+	−	−	+	−	+	是	IGHV3-30*03	TP53/SPEN/KMT2D/FAT1^a^
15	+	+	NA	NA	NA	NA	−	+	否	IGHV3-33*01	NA
16	+	+	−	−	−	−	−	+	否	IGHV4-34*01	KMT2D/SF3B1/NOTCH1/TET2/TNFRSF10B/PTPRD/KLHL6^a^
17	+	−	+	−	−	−	−	+	否	IGHV4-34*01	TP53/NOTCH1^a^
18	+	−	+	−	−	−	−	+	是	IGHV3-15*01	MYD88^a^
19	+	+	−	−	−	−	−	+	否	IGHV4-39*01	SF3B1/NOTCH1/XPO1^a^
20	+	+	−	−	−	−	−	+	否	IGHV1-2*04	KMT2D^a^

注：IGHV：免疫球蛋白重链可变区；CLL：慢性淋巴细胞白血病；+：阳性；−：阴性；NA：无数据；^a^使用72种白血病/淋巴瘤基因检测组合；^b^使用41种白血病/淋巴瘤基因检测组合

4. 分子遗传学结果：除例3因免疫球蛋白重链可变区（IGHV）重排检测阴性，无法进一步检测外，其余19例患者IGHV体细胞突变状态及基因使用情况见表3。可观察到较高比例的未突变IGHV发生率（89％，17/19），且存在IGHV4-39的偏向使用（41％，7/17），其次是IGHV4-34（29％，5/17），这两种基因的使用占所有检测病例的70％。而对于CLL患者，IGHV未突变的发生率为100％（9/9），存在IGHV4-39的偏向使用（56％，5/9）。对17例患者进行二代测序（NGS）检测，其中12例使用72种白血病/淋巴瘤基因检测组合，5例使用41种基因检测组合。14例（82％）检出一种或多种基因突变，共涉及25种基因（表3），其中发生频率最高的基因突变是NOTCH1（6例），其次是SF3B1（4例）、KMT2D（3例）。高频突变、FISH结果和IGHV突变结果见图1。基因突变及染色体异常在CLL组（例1～10）与非CLL组（例11～20）大致呈均匀分布，个别基因如KMT2D突变在非CLL组有倾向性分布。

**图1:**
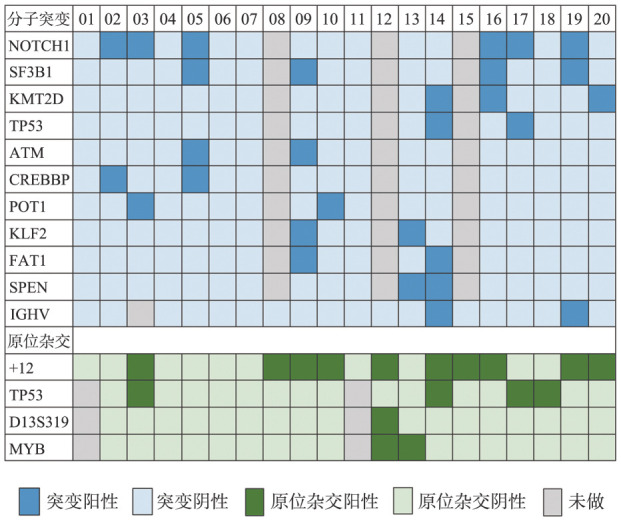
20例伴t（14;19）患者的常见基因突变及FISH异常情况

## 讨论

将本组病例与此前国外报道的t（14;19）病例进行综合分析[3]–[5],[7]–[8]，发现这类疾病大多具有以下共同点：①淋巴细胞增多、淋巴结肿大；②高频率+12和未突变IGHV；③CLL病例有非典型形态及免疫表型报告；④常有侵袭性临床病程，TTT明显缩短。除上述发现外，我们首次发现了高比例（61％）的EB病毒血症及CLL系列中较高的β_2_-微球蛋白水平（平均值3.85 mg/L）。Richter综合征可能由病毒（如EB病毒）感染引发，且往往提示预后不良[9]–[10]，提示伴t（14;19）CLL患者的高EB病毒感染率与Richter转化之间是否存在一定的联系。

既往研究显示，t（14;19）患者中男性占优势，尤其是在+12病例中[4],[11]，而在本组研究中男性和女性的发病率相近（50％对50％）。13q14缺失是常规CLL中最常见的细胞遗传学异常，发生在约55％的患者中，且作为唯一异常时与预后良好相关[12]，但在我们的研究中，这一比例仅为11％（CLL组）和12％（所有病例）。11q23涉及ATM基因，发生在约18％的CLL患者中[12]，但在本组研究检测的17例患者中无ATM缺失者。

Haferlach等[13]报道，涉及14q32/IGH的易位与较短的OS时间相关，是CLL的不良预后因素。此后，Nguyen-Khac等[1]比较IGH两个最常见的伙伴基因BCL2（18q21）和BCL3（19q13），发现后者较前者更具侵袭性。最近一项关于CLL中IGH对手基因的研究也表明，IGH/BCL2与较低的IGHV突变频率相关，并伴有BCL2和IGLL5等突变，而其他非BCL2对手基因CLL患者（IGHR-CLL）的基因突变以NOTCH1、SF3B1和TP53等多见且具有较短的治疗等待期[14]。在本组病例中NGS同样显示了高频率的NOTCH1和SF3B1突变，是CLL预后不良的分子标志物[15]。9例行NGS的伴t（14;19）CLL患者中，3例（33％）患者有NOTCH1突变，此比例显著高于我们既往报道的中国CLL患者中的8.2％和文献报道的11％[16]–[17]。

很多研究显示NOTCH1突变与未突变IGHV及+12显著关联，近40％的NOTCH1突变患者携带+12[18]–[20]。+12和NOTCH1突变被认为表征从属于8号亚组的IGHV4-39 CLL，是一类具有较高Richter转化风险的CLL[21]–[22]。与此相印证，在本组20例t（14;19）患者中有2例发生了此种临床转化：例19具有+12、NOTCH1突变和未突变的IGHV4-39，例12携带+12和未突变的IGHV4-39，但遗憾的是没有标本用于NGS检测以确定NOTCH1突变状态。由于独特的生物学特征，如高频发生的NOTCH1突变、未突变IGHV4-39、+12及EB病毒阳性率，我们应密切关注此类t（14;19）阳性CLL的Richter转化风险。

t（14;19）（q32;q13）IGH/BCL3及其变体t（2;19）（p12;q13）IGK/BCL3和t（19;22）（q13;q11）IGL/BCL3均涉及BCL3基因（IκB蛋白家族成员），因BCL3被鉴定为CLL中的原癌基因并在调节NF-κB信号传导中发挥核心作用，近年来关于BCL3作用的研究逐渐增多，有研究提出抑制NF-κB通路可能在诱导肿瘤细胞凋亡或生长抑制中发挥作用[23]–[24]。对BCL3及NF-κB通路的深入研究可能使t（14;19）或其他BCL3基因相关疾病患者获益。
